# Bidirectional association between zinc and liver cirrhosis: evidence from mendelian randomization and clinical validation

**DOI:** 10.3389/fgene.2026.1833546

**Published:** 2026-07-15

**Authors:** Liu-dan Liang, Ying Yang, Qi-wen Huang, Chen-yi Huang, Bing-fang Lu, Jian-nan Lv, Wei Wei, Ying Li, Hao Wang, Hong-ling Yuan, Qian Li, Ji-ze Huang, Li Li, Mei-jin Huang, Feng-Lian Deng, Li-mei Liang

**Affiliations:** 1 Department of Infectious Diseases, Affiliated Hospital of Youjiang Medical University for Nationalities, Baise, Guangxi, China; 2 Guangxi Clinical Medical Research Center for Hepatobiliary Diseases, Baise, Guangxi, China; 3 Youjiang Medical University for Nationalities, Baise, Guangxi, China; 4 Tianlin County People’s Hospital, Baise, Guangxi, China; 5 Tiandong County People’s Hospital, Baise, Guangxi, China; 6 School of Medical Technology and Artificial Intelligence, Youjiang Medical University for Nationalities, Baise, Guangxi, China

**Keywords:** calcium, liver cirrhosis, mendelian randomization, metal elements, zinc

## Abstract

**Background:**

It remains unclear whether low blood zinc levels in cirrhosis patients are a cause or a consequence of the disease.

**Methods:**

Seven blood metals (calcium, iron, magnesium, phosphorus, copper, selenium, zinc) were assessed using two-sample Mendelian randomization (MR). We performed reverse MR analysis on the positive results and subsequently evaluated the zinc findings in a cohort of 137 cirrhosis patients.

**Results:**

MR analysis revealed that higher genetically predicted zinc levels were associated with an increased cirrhosis risk (OR = 1.24, 95% CI = [1.04, 1.46], p = 0.014). No significant associations were found for other metals. Reverse MR analysis revealed no reverse causality between liver cirrhosis and zinc. In the clinical cohort, 60.5% of patients had hypozincemia, and zinc levels declined progressively with worsening Child-Pugh score (r = – 0.615, p < 0.01). Additionally, a positive correlation was observed between serum zinc levels and albumin (ALB). In contrast, zinc levels were inversely associated with total bilirubin (TBIL), prothrombin time (PT), and international normalized ratio (INR).

**Conclusion:**

Genetically higher zinc levels predispose individuals to cirrhosis, whereas advanced cirrhosis induces zinc deficiency. These bidirectional findings suggest that zinc exerts opposite effects at different disease stages.

## 1 Introduction

Liver cirrhosis accounts for more than a million deaths each year. Hepatitis, alcohol, and non-alcoholic fatty liver disease (NAFLD) explain most cases; however, the etiology of some cases remains unclear. Emerging evidence suggests that metal homeostasis may be involved, although the causal relationship remains unclear (Nangliya et al., 2015; Llibre-Nieto et al., 2021). Therefore, elucidating the causal association between trace metal elements and liver cirrhosis is important for preventing and managing this condition.

Blood essential metal elements are fundamental to maintaining human physiological functions (Zoroddu et al., 2019). The liver is the central organ for metal metabolism and storage, which is intricately linked to metal homeostasis (Schuppan and Afdhal, 2008; Krebs and Hambidge, 2001). The relationship between zinc and liver cirrhosis is complex and exhibits a mutually interactive two-way association. Specifically, zinc dyshomeostasis can serve as both an outcome and a contributing factor in the progression of liver cirrhosis. On the one hand, numerous observational studies have reported that hypozincemia is prevalent in patients with cirrhosis. This zinc deficiency is hypothesized to be a secondary outcome of impaired hepatic synthetic function and intestinal malabsorption, and it is closely linked to malnutrition, immune dysfunction, and poor prognosis (Mohammad et al., 2012; Grüngreiff et al., 2016). On the other hand, laboratory experiments and animal models have also demonstrated that excessive zinc exposure may exacerbate liver fibrosis by inducing reactive oxygen species (ROS) generation and activating inflammatory signaling pathways (Sharma et al., 2012a). The mechanism underlying this potential “bidirectional” role of zinc remains unclear. The central controversy is whether zinc dysregulation is an etiological factor or an outcome of the disease. Traditional surveys face drawbacks due to confounding factors and reverse causality, which preclude definitive causal inference. MR is an epidemiological technique that uses genetic variation to infer causal relationships. Single-nucleotide polymorphisms (SNPs) that are strongly related to exposure factors are chosen as instrumental variables in this approach. This method simulates a quasi-randomized research design to effectively adjust for confounding biases and reverse causality concerns (Yuan et al., 2024). Three fundamental assumptions underpin the main principle of genetic variation. These assumptions are as follows: (1) genetic variation is strongly associated with susceptibility; (2) genetic variation is independent of confounding factors; and (3) genetic variation affects the outcome solely through susceptibility (Davies et al., 2018). No study has systematically tested multiple blood metals for causality in cirrhosis using rigorous MR, and then validated the findings in patients.

Given this background, this study first employed a two-sample MR design utilizing rigorously selected genetic instrumental variables. We comprehensively evaluated the causal associations between seven key blood metals (calcium, iron, magnesium, phosphorus, copper, selenium, and zinc) and the risk of liver cirrhosis. The primary focus was on validating the robustness of the zinc findings. To bridge the gap between causal inference and clinical practice, we enrolled 137 cirrhosis patients and analyzed correlations of serum zinc levels with Child-Pugh score and key hepatic function indicators. We combined genetic causality testing with real-world patient data to solve the zinc paradox.

## 2 Methods

### 2.1 Study design

The two-sample MR study design was adopted to estimate the causal link between blood essential metal elements (calcium, iron, magnesium, phosphorus, copper, selenium, and zinc) and liver cirrhosis. This approach relies on three core assumptions: (1) genetic instruments are strongly associated with the exposure; (2) genetic instruments affect the outcome exclusively through the exposure; (3) genetic instruments are independent of any known confounders of the exposure-outcome association. Meanwhile, reverse MR analyses were further performed to assess the potential causal effect of the outcome on the exposure.

### 2.2 Instrumental variables (IVs) and outcome genome-wide association studies (GWAS)

In this study, a total of 7 kinds of blood essential metal elements were obtained from previously published GWAS studies (Table 1). To fulfill three main assumptions in MR analysis, we applied two sets of quality control steps for selection of IVs for each element. For inclusion criteria: (1) SNPs were identified at P < 5.0 × 10^–8^ (relaxed to p < 5 × 10^−6^ for metals with fewer than three SNPs); and (2) when calculating linkage disequilibrium (LD), independent SNPs were selected by clumping at r^2^ < 0.001 (window = 5,000 kb); For exclusion criteria: (1) SNPs missing in the outcome GWAS dataset were excluded; and (2) no proxy SNPs were used. To reduce potential bias arising from missing IVs, the exposure and outcome datasets were harmonized prior to the clumping step. To assess the potential impact of weak instrument bias on causal effect estimates, F-statistics was assessed, with $R^{2}$ representing the variance explained by the IVs (Papadimitriou et al., 2020; Burgess et al., 2011). To minimize the sample overlap between the exposure and the outcome, we downloaded genetic results for liver cirrhosis from the FinnGen consortium, containing 1,266 cases and 407,801 controls (Data Freeze 10).

**TABLE 1:** Characteristics of selected GWAS in this study.

Trait	References	Ancestry	Sample size	Numbers of SNPs
Essential metal elements				
Calcium	Barton et al. (2021)	European	400,792	259
iron	Benyamin et al. (2014)	European	23,986	3
zinc	Evans et al. (2013)	European	2,603	7
copper	Evans et al. (2013)	European	2,603	6
selenium	Evans et al. (2013)	European	2,874	6
magnesium	Meyer et al. (2010)	European	15,366	5
phosphorus	Kestenbaum et al. (2010)	European	16,264	5
Outcome				
liver cirrhosis	FinnGen	European	1,266 cases/407,801 controls	​

Table 1. Characteristics of GWAS for blood essential metal elements and liver cirrhosis included in the MR analysis. This table summarizes key details of the GWAS datasets used, including 7 blood essential metal elements (calcium, iron, magnesium, phosphorus, copper, selenium, and zinc) as exposures and liver cirrhosis as the outcome. Smaller GWAS for some metals reflect the limited availability of published data. SNPs, single-nucleotide polymorphisms.

### 2.3 Baseline characteristics of the study cohort

A total of 137 patients [89 males (65.0%) and 48 females (35.0%); 50.4 ± 9.3 years] with liver cirrhosis were consecutively enrolled at the Affiliated Hospital of Youjiang Medical University for Nationalities from January 2023 to January 2025. The etiological distribution was as follows: hepatitis B virus (HBV)-related cirrhosis (n = 91), hepatitis C virus (HCV)-related cirrhosis (n = 12), alcoholic cirrhosis (n = 15), schistosomal cirrhosis (n = 5), and mixed-etiology cirrhosis (n = 12). Among these 12 patients with mixed-etiology cirrhosis, five had HBV/HCV co-infection and seven had HBV infection combined with alcoholic cirrhosis. All patients fulfilled the diagnostic and exclusion criteria for liver cirrhosis as defined by the Guidelines for the Prevention and Treatment of Chronic Hepatitis B (2010) (Society Of Infectious Diseases CMA and Society Of Hepatology CMA, 2019). The analysis was approved by the Ethics Committee of the hospital, and written informed consent was obtained from each patient or their legal guardian.

### 2.4 Sample collection and laboratory measurements

Upon hospital admission and prior to treatment initiation, venous blood samples were collected from all enrolled patients. Serum zinc concentrations and routine biochemical parameters [including total bilirubin (TBIL), prothrombin time (PT), albumin (ALB), and international normalized ratio (INR)] were determined. Neurological status was assessed clinically to stage hepatic encephalopathy, while abdominal ultrasonography was performed to evaluate the presence and severity of ascites. Liver function was graded according to the Child-Pugh scoring system: Class A (5–6 points), Class B (7–9 points), and Class C (D'Alessio et al., 2022). All laboratory measurements were conducted in accordance with standardized protocols in the hospital’s central laboratory, ensuring consistency and reliability of the results.

### 2.5 Statistical analysis

The primary two-sample MR analysis was performed using the inverse variance weighted (IVW) method as the main approach. Sensitivity analyses included the weighted median, Mendelian Randomization-Egger regression (MR-Egger) with simulation extrapolation (SIMEX) correction for dilution bias, Mendelian Randomization Pleiotropy RESidual Sum and Outlier (MR-PRESSO), and leave-one-out. Additionally, reverse MR was performed to assess whether the outcome influences the exposure. All analyses were conducted using the R package Two Sample MR (version 0.5.8). For the clinical cohort, statistical analyses were performed using SPSS (Version 26.0). Continuous variables were expressed as mean ± standard deviation (SD), and categorical variables as counts and percentages. Comparisons across Child-Pugh classes were conducted using one-way analysis of variance (ANOVA). Pearson correlation coefficients were calculated to assess associations between serum zinc concentrations and liver function indicators. All statistical tests were two-tailed, and a p-value <0.05 was considered statistically significant.

## 3 Results

### 3.1 Selection of genetic variants

The genetic instruments selected for blood essential metal elements are summarized in Table 1, with additional information in Supplementary Table S1. Specifically, 259, 3, 5, 5, 6, 6, and 7 associated variants were identified for calcium, iron, magnesium, phosphorus, copper, selenium, and zinc, respectively. The F-statistics for all instruments were substantially greater than 10, suggesting that weak instrument bias was unlikely to materially affect the MR estimates (Supplementary Table S2). Due to the lack of sufficient IVs, the p-value threshold for zinc, copper, selenium, and magnesium was relaxed to ×5 10^−6^.

### 3.2 MR analysis

**FIGURE 1:**
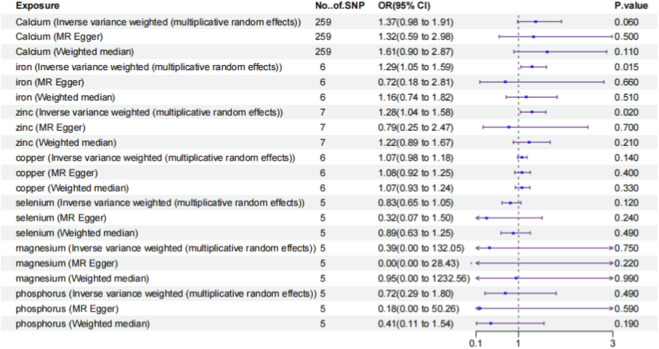
Forest plot of MR estimates for the causal associations between seven essential blood metal elements and the risk of liver cirrhosis. Blue dots represent the MR effect estimates, and purple bars represent the corresponding 95% confidence intervals. An OR > 1 indicates an increased risk, and an OR < 1 indicates a decreased risk. MR, Mendelian randomization; CI, confidence interval; OR, odds ratio.

The IVW method demonstrated a significant positive association between genetically predicted serum zinc levels and the risk of liver cirrhosis (OR = 1.24; 95% CI (1.04, 1.46), p = 0.014; Table 2). The weighted median estimator yielded a consistent direction of effect, although the association did not reach statistical significance (OR = 1.15; 95% CI = (0.90, 1.47), p = 0.24) (Figure 1). For zinc, violation of the no measurement error (NOME) assumption was observed ( $I_{GX}^{2}$ = 61.10%), indicating dilution bias in MR-Egger regression. The SIMEX-corrected MR-Egger estimate was directionally consistent but had a wider CI (OR = 1.12; 95% CI = [0.90, 1.38], p = 0.352), reflecting reduced power after correction for dilution. For calcium, initial IVW and MR-Egger analyses suggested a potential association with cirrhosis risk. However, subsequent sensitivity analyses revealed substantial heterogeneity (Cochran’s Q test, p < 0.05), and the MR-PRESSO global test identified two outlier SNPs. After removing these outliers, the association disappeared, indicating that the initial signal was likely due to genetic pleiotropy rather than a true causal effect. No significant causal associations were identified for iron, magnesium, phosphorus, copper, or selenium across all MR methods, underscoring the specificity of the zinc finding (Supplementary Table S6).

**TABLE 2:** MR estimates of calcium and zinc in liver cirrhosis.

Exposure	MR method	No. Of SNP	OR (95%CI)	P-value
Calcium	Inverse variance weighted (multiplicative random effects)	259	1.37 (0.98–1.91)	0.062
Calcium	MR Egger	259	1.32 (0.59–2.98)	0.50
Calcium	Weighted median	259	1.61 (0.90–2.87)	0.110
Zinc	Inverse variance weighted (multiplicative random effects)	7	1.24 (1.04–1.46)	1.40E-02
Zinc	SIMEX-MR Egger	7	1.12 (0.90–1.38)	0.352
Zinc	Weighted median	7	1.15 (0.91–1.47)	0.24

Table 2. MR estimates of the causal effects of blood essential metal elements on liver cirrhosis. This table presents the causal effect estimates of calcium and zinc on liver cirrhosis. SNPs, single-nucleotide polymorphisms. OR, odds ratios; CI, confidence intervals.

### 3.3 Sensitivity analyses and leave-one-out analysis

In the sensitivity analyses, the p values for Cochran’s Q test were greater than 0.05 in both the IVW and MR-Egger models for all essential metal elements except calcium. This suggests no substantial heterogeneity among the instrumental variables for most exposures (Supplementary Table S3). For calcium, however, significant heterogeneity was detected, indicating potential instability in the initial MR estimate. The MR-Egger intercepts were close to zero for all essential metal elements, providing no evidence of directional horizontal pleiotropy (Supplementary Table S4). For all essential metal elements, the P values from the MR-PRESSO global tests were greater than 0.05, providing no evidence of any outlier SNPs. (Supplementary Table S5). This finding suggests that the initial calcium-related signal was likely influenced by outlier-driven pleiotropy. For zinc, the leave-one-out analysis showed that the risk estimates remained directionally consistent after sequentially excluding each SNP, meaning that no single SNP drove the overall association. This supports the stability of the zinc-related MR finding (Supplementary Figure S1).

### 3.4 Reverse MR analysis outcomes

The IVW analysis indicated no genetically mediated causal association between cirrhosis susceptibility and serum zinc levels (OR = 1.01; 95% CI = [0.94, 1.08], p = 0.84). This null causal relationship was consistently verified using the weighted median method (OR = 1.03; 95% CI = [0.94, 1.12], p = 0.586) and SIMEX-corrected MR-Egger regression (OR = 1.04; 95% CI = [0.87, 1.24], p = 0.0.655). No obvious horizontal pleiotropy was observed, as reflected by the non-significant MR-Egger intercept (p = 0.52) and global MR-PRESSO test result (p = 0.78). Leave-one-SNP sensitivity analysis demonstrated that no individual genetic variant could dominate the overall null findings. Additionally, all selected instrumental variables had F-statistics above 10, suggesting that weak instrument bias was not evident in the present analysis (Supplementary Table S7).

### 3.5 Clinical characteristics of the cirrhosis cohort

A total of 137 patients with liver cirrhosis were included in the clinical cohort and stratified according to Child-Pugh classification. Specifically, 45 patients were classified as class A, 51 as class B, and 41 as class C. Serum zinc concentrations ranged from 3.2 to 18.2 μmol/L (reference range: 10.8–19.4 μmol/L). Overall, 83 patients (60.5%) had hypozincemia. The prevalence of hypozincemia increased with disease severity: 35.6% in Child-Pugh class A, 62.8% in class B, and 85.4% in class C. Mean serum zinc concentrations differed significantly among the three Child-Pugh groups (p < 0.01). Significant differences were also observed in liver function indicators (including ALB, TBIL, PT, and INR), and all of these indicators varied across Child-Pugh severity categories (p < 0.01) (Table 3).

**TABLE 3:** Comparison of serum zinc, TBIL, ALB, PT, and INR levels among patients with different liver function grades (x ± s).

Group	N	Zinc (umol/L)	TBIL (umol/L)	ALB (g/L)	PT(s)	INR
Group A	45	12.86 ± 4.13	27.24 ± 4.10	38.52 ± 2.28	12.23 ± 1.67	1.04 ± 0.17
Group B	51	9.60 ± 3.60*	36.01 ± 8.52*	31.11 ± 2.04*	14.03 ± 1.62*	1.22 ± 0.16*
Group C	41	7.61 ± 2.37*	57.57 ± 16.31*	24.95 ± 3.44*	18.12 ± 0.92*	1.59 ± 0.12*
F value	​	14.63	92.69	292.39	187.62	145.51
P value	​	0.001	0.001	0.001	0.001	0.001

Table 3. Comparison of serum zinc, TBIL, ALB, PT, and INR levels among patients with different liver function classes (x±s). This table presents the mean ± SD of serum zinc, TBIL, ALB, PT, and INR in cirrhosis patients stratified by Child-Pugh classes (A, B, C). It includes sample sizes, F-values, and p-values to demonstrate statistically significant differences in these indicators across Child-Pugh classes. TBIL, total bilirubin; ALB, albumin; PT, prothrombin time; INR, international normalized ratio. * p < 0.05, compared with Group A.

### 3.6 Correlation analysis between zinc levels and hepatic function indicators

**FIGURE 2:**
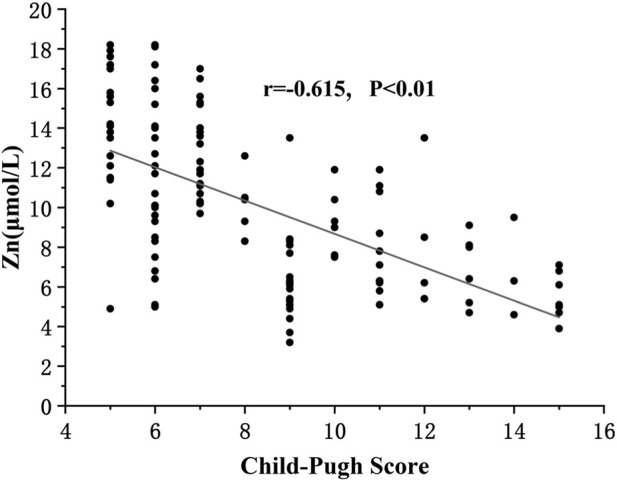
Scatter plot showing the negative correlation between serum zinc levels and Child-Pugh scores in patients with liver cirrhosis. Each point represents an individual patient from the clinical cohort (n = 137). Serum zinc concentration is plotted against the Child-Pugh score. The analysis showed a strong and statistically significant negative correlation (r = −0.615, p < 0.01), indicating that lower serum zinc levels were closely associated with more advanced liver disease.

As indicated by Pearson correlation analysis results, serum zinc levels were negatively correlated with TBIL, PT, and INR, and positively correlated with ALB (Table 4). Notably, serum zinc concentrations showed a strong negative correlation with Child-Pugh scores (r = −0.615, p < 0.01), indicating that lower zinc levels were closely associated with more severe hepatic dysfunction (Figure 2).

**TABLE 4:** Correlation analysis of serum zinc levels with TBIL, ALB, PT, and INR, stratified by disease severity group.

Group	TBIL (umol/L)		ALB (g/L)		PT (s)		INR	
	r value	p value	r value	p value	r value	p value	r value	p value
Group A	−0.49	0.001	0.75	0.001	−0.7	0.001	−0.68	0.001
Group B	−0.51	0.001	0.35	0.01	−0.61	0.001	−0.57	0.001
Group C	−0.49	0.001	0.47	0.002	−0.44	0.004	−0.41	0.009

Table 4. Correlation analysis of serum zinc levels with TBIL, ALB, PT, and INR, stratified by disease severity group. This table presents Pearson correlation coefficients and corresponding p-values, illustrating the associations between serum zinc levels and TBIL, ALB, PT, and INR in cirrhosis patients grouped by Child-Pugh classes (A, B, C). It highlights consistent directional correlations (negative for TBIL, PT, and INR; positive for ALB) across all disease severity groups. TBIL, total bilirubin; ALB, albumin; PT, prothrombin time; INR, international normalized ratio.

## 4 Discussion

This study employed a two-sample MR framework to systematically evaluate the causal associations between seven essential blood metals and liver cirrhosis. The results demonstrated that zinc was notably associated with cirrhosis risk, whereas the initial signal for calcium was attributable to pleiotropy, as shown in the supplementary analyses. No causal effects were observed for the other metals. Notably, in this study, no causal relationship was identified between peripheral blood iron levels and liver cirrhosis, which is inconsistent with previous observational evidence of tissue iron overload (Wang et al., 2022). Clinical validation further confirmed that serum zinc concentrations were markedly reduced in patients with cirrhosis and declined progressively with disease severity. Collectively, our findings provide novel evidence supporting a bidirectional relationship between zinc and liver cirrhosis. Genetically elevated zinc levels may contribute to disease onset, while progressive cirrhosis may lead to secondary zinc deficiency. These observations underscore the intricate crosstalk between micronutrient metabolism and the pathogenesis of chronic liver disease. Furthermore, they suggest that zinc status should be closely monitored and managed in a stage-specific manner during cirrhosis care.

Zinc is a first-row transition metal and an essential micronutrient; it is widely distributed within cells, including the cytoplasm, mitochondria, nucleus, and Golgi apparatus. It is a key structural component of more than 3,000 transcription factors and acts as a core component or cofactor for over 300 enzymes (Coradduzza et al., 2024). Furthermore, zinc plays a pivotal role in maintaining normal cellular function by regulating diverse biological processes, including immune responses, transcription factor activity, cell differentiation and proliferation, nucleic acid synthesis and repair, enzymatic activity, signal transduction, and cell structure and membrane stability (Jiang et al., 2022). Although zinc is generally considered relatively safe, disruption of zinc homeostasis, either through deficiency or excess, can have significant effects on human health (Plum et al., 2010). Previous studies have shown that low zinc concentrations are associated with increased oxidative stress, impaired immunity, cardiovascular complications, and the development of atherosclerosis (Wani et al., 2017). Conversely, the adverse effects of excessive zinc intake are less well documented, but they have been reported to disrupt the balance of other essential nutrients (including iron, calcium, selenium, nickel, phosphorus, and copper) (El et al., 2008). For example, excess zinc may enhance IL-18-mediated inflammation, thereby increasing the risk of pulmonary infections (Yang et al., 2021), chronic obstructive pulmonary disease (COPD) (Huang et al., 2025), and chronic renal failure (Ay et al., 2022).

As the central organ for metabolic regulation and detoxification, the liver plays a pivotal role in zinc metabolism (Coni et al., 2021). Zinc homeostasis is essential for maintaining normal hepatic function. A previous study has shown that zinc deficiency can promote liver fibrosis, whereas zinc supplementation may protect against liver fibrosis progression (Nishikawa et al., 2022). However, excessive zinc accumulation may also induce hepatic dysfunction by interfering with key enzymatic activities (Mizari et al., 2012; Ding et al., 1998). In a study of 4,138 United States adults aged 19–50 years, serum zinc levels are positively associated with elevated alanine transaminase (ALT) (Hu et al., 2021). Similarly, a study of adolescents in Taiwan, China, has also found that higher dietary zinc intake is associated with increased serum ALT levels (Bai et al., 2017). Additionally, a study in occupational populations has shown that zinc exposure among smelter workers can cause marked alterations in serum phospholipid metabolic pathways, accompanied by elevated ALT levels (Wang et al., 2024). These observations suggest that excessive zinc exposure may be closely linked to liver injury. However, the specific mechanisms by which long-term, high-concentration zinc exposure affects the human liver remain unclear. This study found a robust causal association between genetically predicted serum zinc levels and liver cirrhosis. In the IVW analysis, a one–SD increase in serum zinc was associated with a 24% higher risk of cirrhosis. To assess reverse causation, we performed reverse MR, treating liver cirrhosis as the exposure and metal levels as the outcome. The analysis revealed no reverse causality between liver cirrhosis and zinc. This finding is consistent with existing basic research evidence. At physiological concentrations, zinc serves as an indispensable cofactor for antioxidant enzymes, including superoxide dismutase (SOD), which helps scavenge ROS. However, excessive zinc is detrimental, as it can paradoxically induce oxidative stress (via the Fenton reaction), trigger mitochondrial dysfunction, and ultimately lead to hepatocyte death (Sharma et al., 2012b; Stehbens, 2003).

In our clinical cohort of 137 patients with liver cirrhosis, hypozincemia was detected in 60.5% of participants, and its prevalence increased progressively with worsening Child-Pugh class. Serum zinc concentrations were negatively correlated with TBIL, PT, and INR levels, but positively correlated with ALB levels. Furthermore, a strong negative correlation was observed between serum zinc levels and Child-Pugh scores. Consistently, Semeya et al. (2025) have shown that lower serum zinc levels are associated with higher Child-Pugh scores, lower ALB levels, and greater severity of hepatic encephalopathy in patients with cirrhosis. The mechanisms by which cirrhosis leads to a low-zinc state are likely multifactorial. First, the liver is the primary site for the synthesis of zinc-binding proteins, particularly ALB. Extensive hepatocyte damage reduces ALB synthesis, thereby decreasing zinc-binding capacity and promoting zinc loss (Katayama, 2020). Second, cirrhosis-associated portal hypertension can cause intestinal congestion and edema, impairing mucosal absorption and reducing intestinal zinc uptake (Stamoulis et al., 2007). Third, portosystemic shunting in cirrhosis may increase urinary zinc excretion (Schölmerich et al., 1983). Importantly, hypozincemia is not merely a passive outcome of cirrhosis; instead, it may further exacerbate disease progression (Ullah et al., 2023). Zinc deficiency can induce oxidative tissue injury and modulate specific hepatic signaling pathways (Oteiza and Mackenzie, 2005). Additionally, it may also induce oxidative stress (Powell, 2000), increase susceptibility to hepatitis, weaken the protective acute-phase response, and promote lipid peroxidation. Zinc deficiency disrupts redox homeostasis and impairs the function of redox-sensitive transcription factors; this consequently affects cellular function, proliferation, and survival (DiSilvestro, 2000; Prasad and Bao, 2019)—processes that are particularly critical for hepatic regeneration. Moreover, zinc can ameliorate palmitate-induced endoplasmic reticulum stress and impaired autophagic flux, thereby protecting hepatocytes from various endoplasmic reticulum stress-related injuries (Bozym et al., 2010). Therefore, liver cirrhosis and hypozincemia may form a mutually reinforcing vicious cycle. These findings suggest that active monitoring and correction of hypozincemia may represent a potential therapeutic strategy to slow disease progression in cirrhosis management.

## 5 Conclusion

This study integrated two-sample MR analysis with clinical cohort validation to investigate the causal role of essential blood metals in liver cirrhosis. Among the seven metals examined, zinc was the only factor that showed consistent evidence of association. Genetic analyses indicated that elevated zinc levels may predispose individuals to the onset of cirrhosis, whereas clinical data revealed that zinc deficiency is highly prevalent in advanced disease and strongly correlated with impaired liver function. These findings provide a novel perspective on the pathogenesis of liver cirrhosis and offer a potential evidence base for its prevention and clinical management. Serum zinc levels may serve as both a genetic risk marker and an indicator for monitoring disease progression.

This study has two main strengths. First, we used large-scale GWAS data from the FinnGen consortium, minimizing bias due to sample overlap. Second, we integrated genetic causal inference with clinical validation. However, this study has several limitations. First, the genetic dataset was restricted to individuals of European ancestry. Second, the clinical cohort was relatively small and single-center. Third, missing instrumental variable SNPs in the outcome datasets may introduce potential bias to the causal estimation results,to mitigate such bias, the instrumental variables were harmonized prior to the clumping step. Additionally, our reliance on serum zinc concentrations may not fully reflect hepatic zinc stores. Future studies should validate these findings in non-European populations and directly assess hepatic zinc levels. Mechanistic investigations are also needed to further elucidate the bidirectional effects of zinc in liver cirrhosis.

## Data Availability

The data analyzed in this study is subject to the following licenses/restrictions: The datasets analyzed during the current study are available from the corresponding author on reasonable request, subject to ethical approval and data access agreements with the original data providers. The clinical phenotypic data are not publicly available due to privacy and ethical restrictions to protect patient confidentiality. Requests to access these datasets should be directed to Liu-dan Liang, 806481476@qq.com.
