# Supplementary figures and images for: Bidirectional association between zinc and liver cirrhosis: evidence from mendelian randomization and clinical validation
Source: Front Genet. 2026 Jul 15;17:1833546. doi: 10.3389/fgene.2026.1833546 (PMC13413658; doi:10.3389/fgene.2026.1833546)

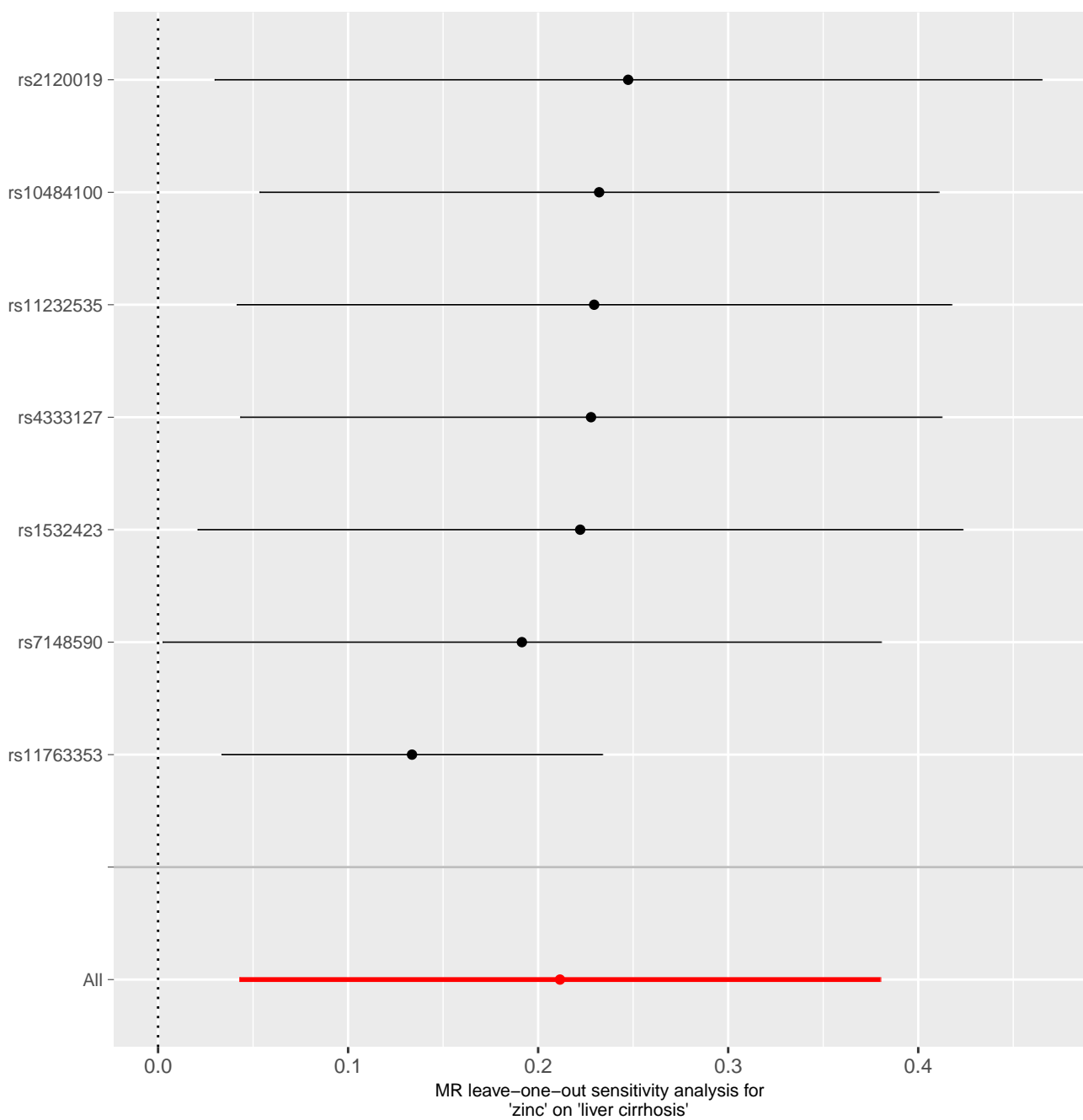

Supplement: Supplementary file 1 [file DataSheet7.pdf]

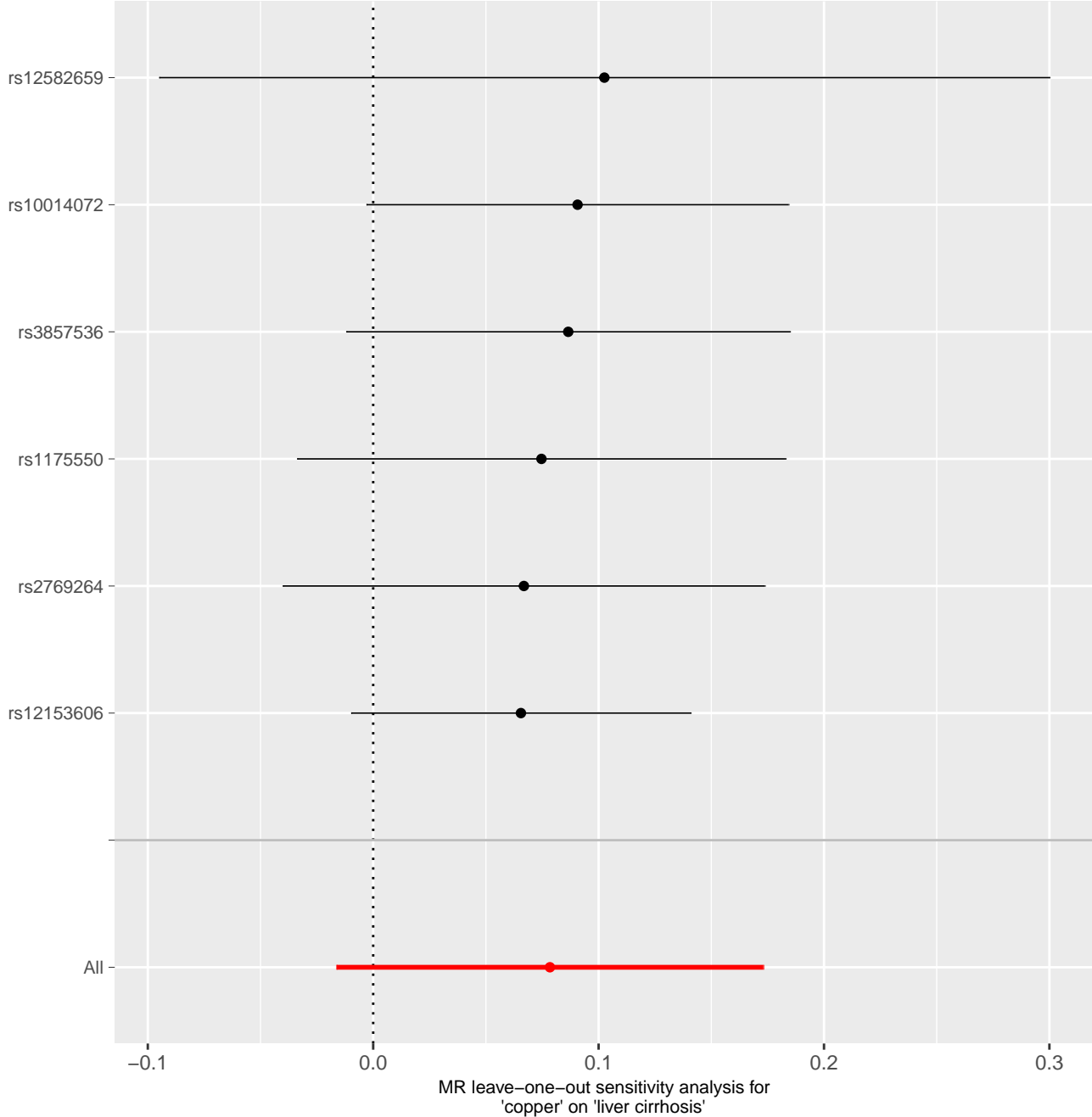

Supplement: Supplementary file 2 [file DataSheet2.pdf]

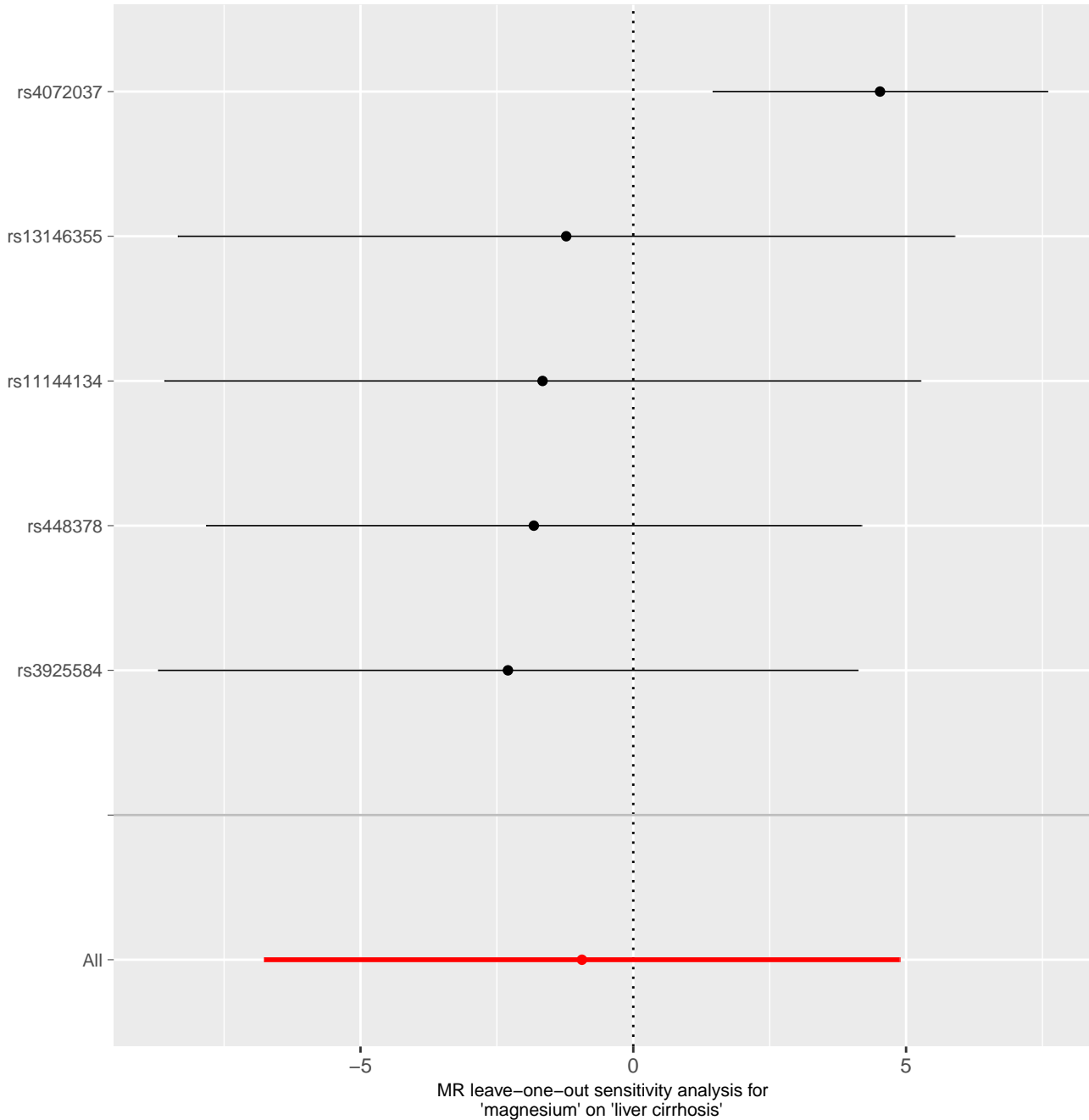

Supplement: Supplementary file 3 [file DataSheet4.pdf]

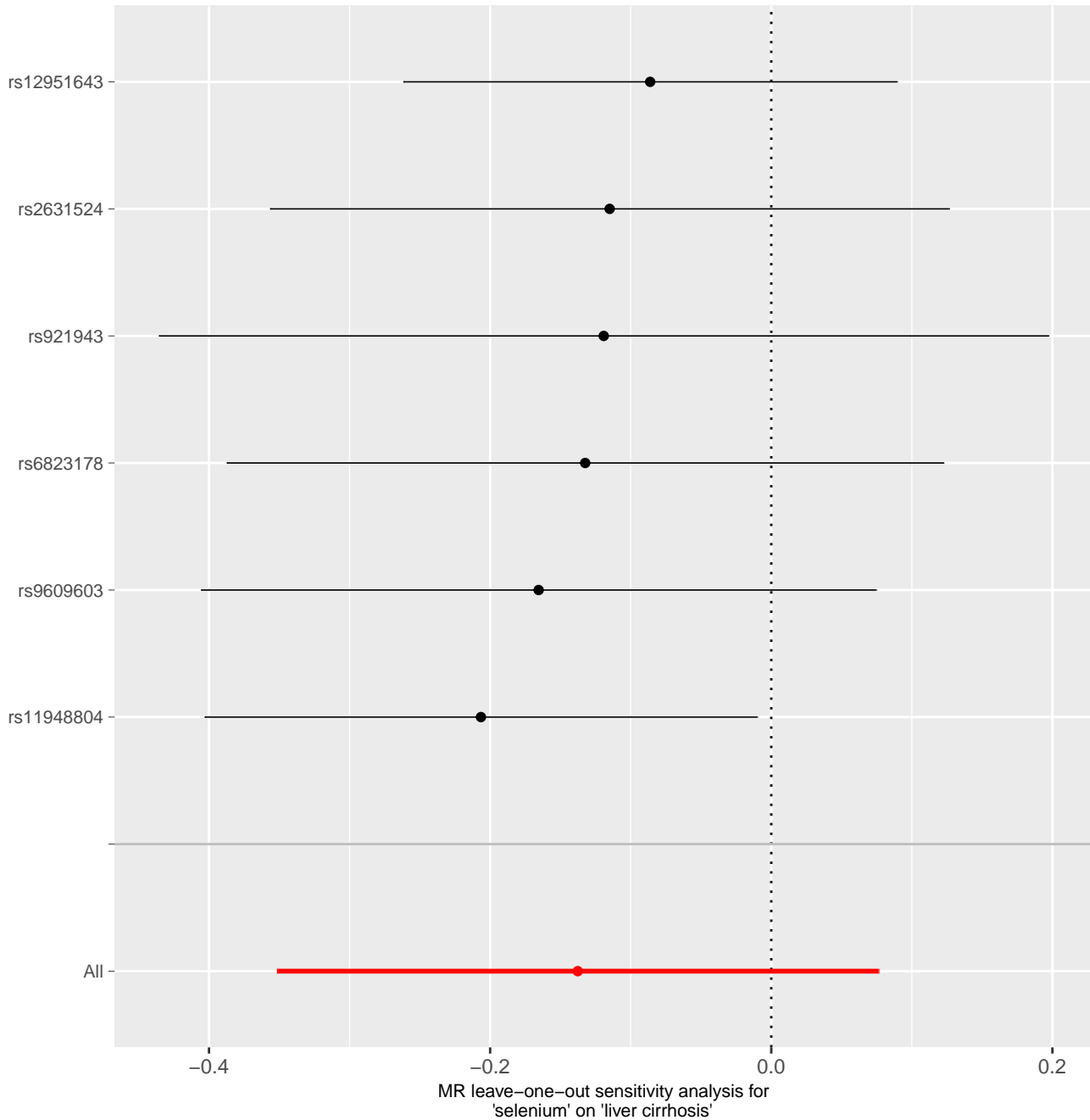

Supplement: Supplementary file 4 [file DataSheet6.pdf]

rs855791

rs1800562

rs1525892

All

-0.5

0.0

0.5

MR leave-one-out sensitivity analysis for  
'iron' on 'liver cirrhosis'

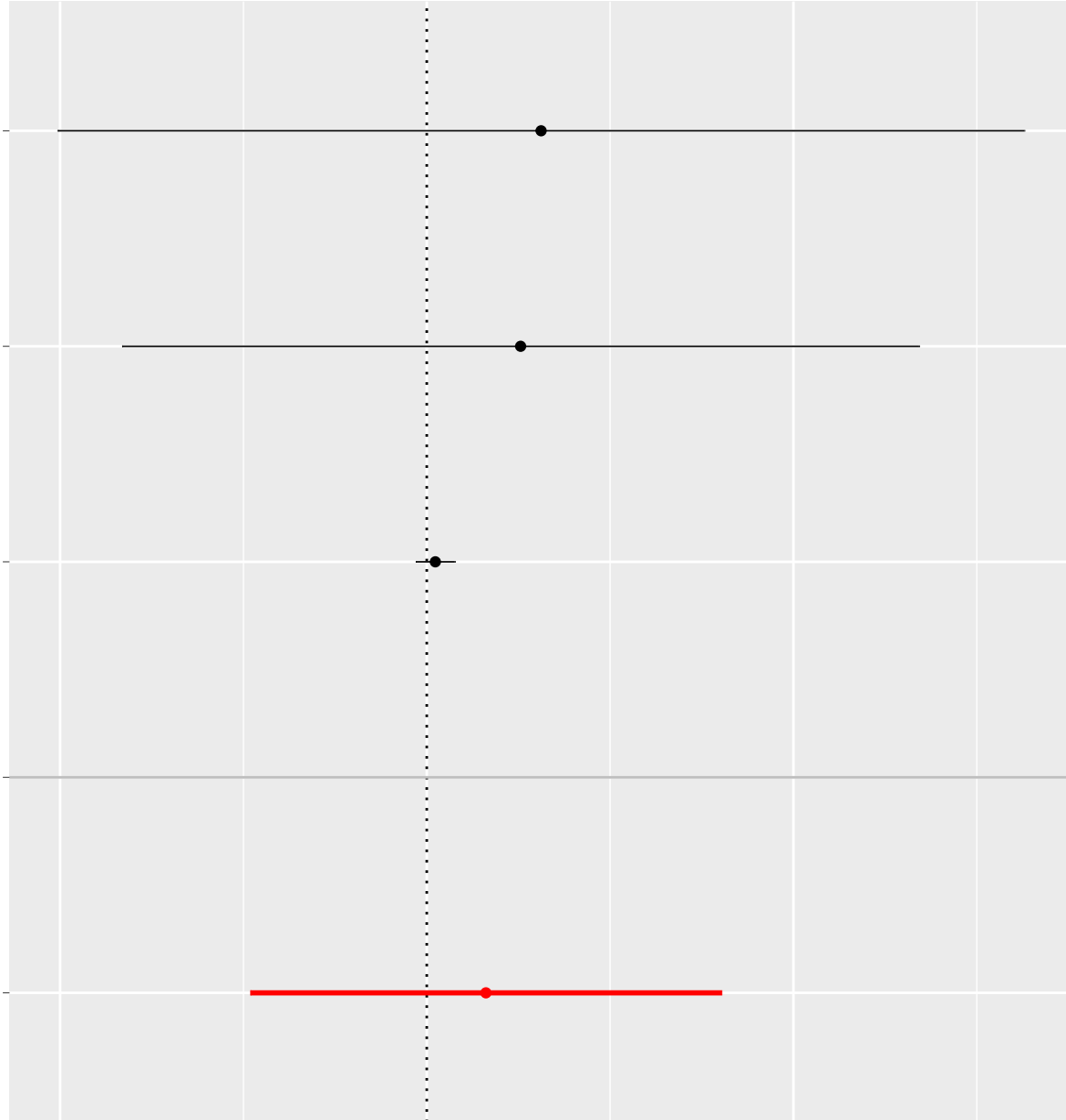

Supplement: Supplementary file 5 [file DataSheet3.pdf]

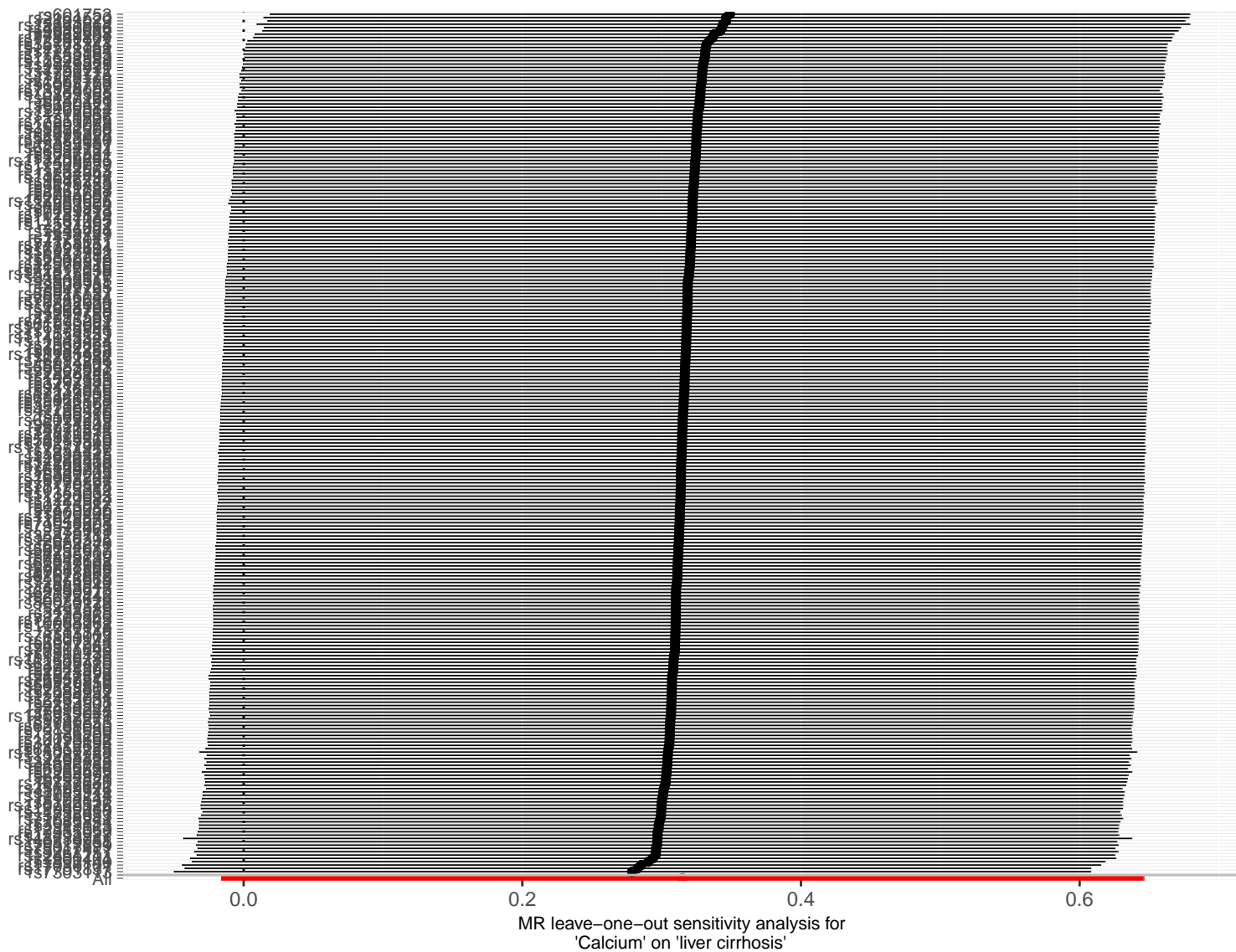

Supplement: Supplementary file 6 [file DataSheet1.pdf]

rs1697421

rs17265703

rs9469578

rs947583

rs2970818

All

-1

0

1

MR leave-one-out sensitivity analysis for  
'phosphorus' on 'liver cirrhosis'

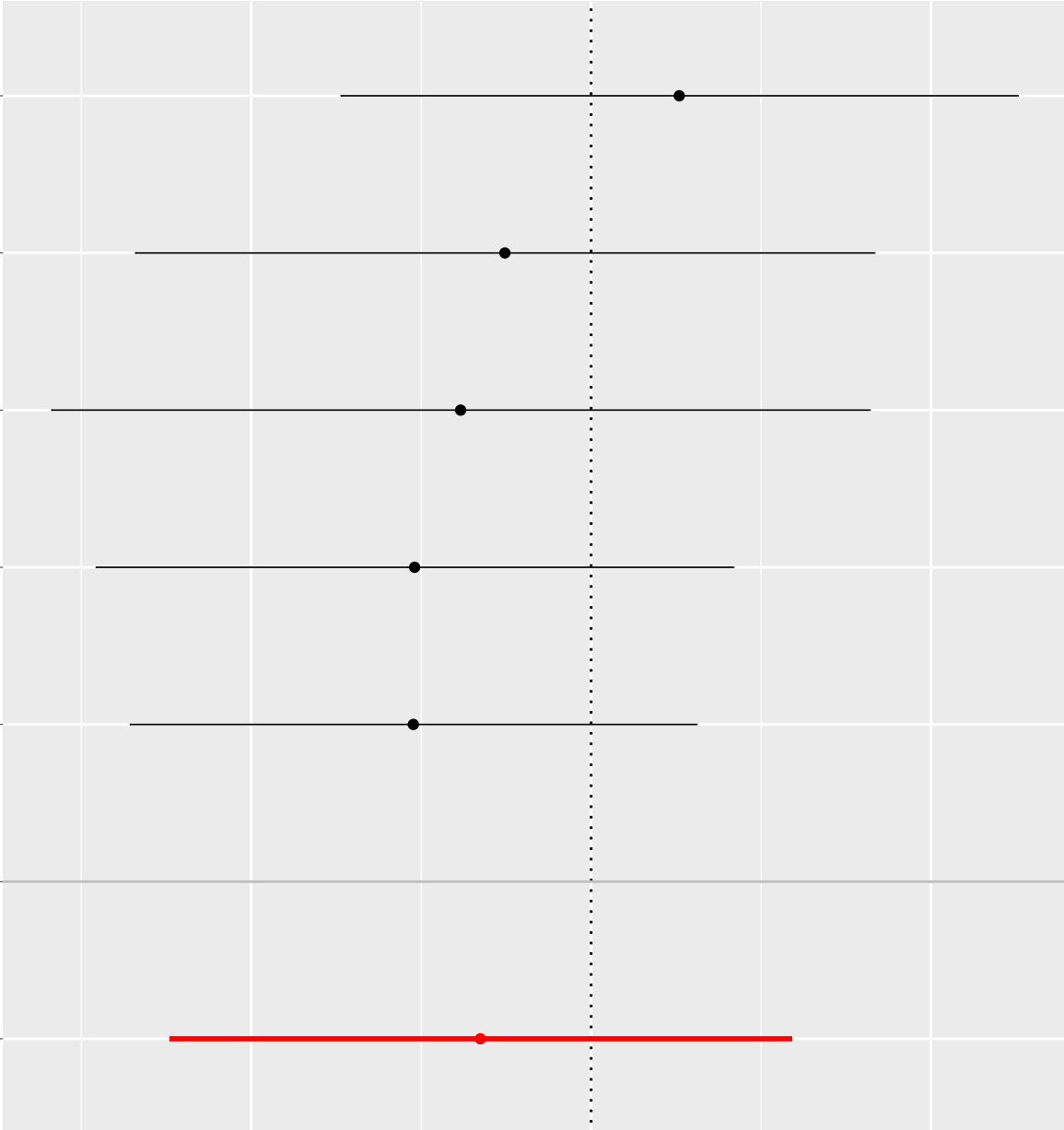

Supplement: Supplementary file 7 [file DataSheet5.pdf]
